# Community‐based care needs for adults with class III obesity before and after tertiary weight management: An exploratory study

**DOI:** 10.1002/osp4.732

**Published:** 2024-01-11

**Authors:** Jillian Termaat, Milan K. Piya, Kate A. McBride

**Affiliations:** ^1^ School of Medicine Western Sydney University Penrith New South Wales Australia; ^2^ South Western Sydney (SWS) Metabolic Rehabilitation and Bariatric Program Camden and Campbelltown Hospitals Camden New South Wales Australia; ^3^ Translational Health Research Institute Western Sydney University Penrith New South Wales Australia

**Keywords:** class 3 obesity, community‐based care, social support, tertiary weight management, transition

## Abstract

**Objective:**

Class 3 obesity (severe obesity) is defined by a body mass index ≥40 kg/m^2^. Tertiary weight‐management programs (WMPs) are hospital‐based multidisciplinary services that aim to support individuals with severe obesity. Severe shortage of WMPs has led to waitlists and pressure on clinicians to discharge patients. Community obesity management often fails to support patients in maintaining weight loss/health gains. This study aimed to explore the needs of patients for community‐based obesity care.

**Methods:**

A qualitative study was undertaken via a tertiary WMP in Sydney, Australia. Semi‐structured interviews/focus groups explored perceptions of purposively sampled patients and their clinicians on the community‐based support needs of people with severe obesity. Data were audio‐recorded, transcribed verbatim, and then thematically analyzed.

**Results:**

Eleven patients and seven clinicians were interviewed. Four themes were identified: the importance of accountability and motivation to maintain weight‐loss/health gains; limitations within community‐based obesity management for those with severe obesity; perspectives on structured community programs for patients transitioning into/out of tertiary WMPs; and impact of mental health, stigma, and social isolation on engagement with community‐based services.

**Conclusions:**

Community‐based programs are needed to support those awaiting access to tertiary WMPs and to help maintain health gains once discharged. Such programs should address issues of social isolation and integrate with current models of tertiary metabolic and primary health care.

## 1 INTRODUCTION

In 2021 in Australia, over a third of all adults (34.6%) were estimated to have obesity, with 80% of the population predicted to meet the definition of clinical obesity (body mass index [BMI] ≥30 kg/m^2^) by 2025.^1^, ^2^ Obesity significantly increases the risk of chronic disease; and reduces wellbeing and quality of life.^3^ Class 3 obesity, or severe obesity, refers to people with a BMI ≥40 kg/m^2^).^4^

There is a greater prevalence of obesity in lower socioeconomic groups, as well as those from cultural and linguistically diverse populations.^1^, ^5^, ^6^ Additionally, many of these populations exist within obesogenic environments, defined in literature as an environment in the home, community, or workplace that promotes gaining weight and hinders weight loss.^7^ There is a scarcity of tertiary weight‐management programs (WMPs) in Australia, leading to a high demand for these services. They provide integrated medical management (including lifestyle change), psychological support, and bariatric surgery for patients with severe obesity.^2^, ^8^ Tertiary WMPs have been shown to reduce acute hospital use in severe obesity, with improved access to such services having significant potential to reduce pressure on hospital capacity and alleviate acute healthcare costs.^9^ These services have limited availability and capacity, with wait times of up to 24 months in Australian settings.^10^ There is a distinct lack of structured services available to assist these complex patients while on waiting lists for, or following completion of, treatment at a WMP.^11^ It is widely accepted that the maintenance of weight‐loss and health gains requires ongoing attention.^12^ This is difficult to achieve in people with severe obesity outside tertiary WMPs, highlighting a care gap within the current system.^11^, ^12^

To date, only very limited research exists on the support necessary for patients returning to community‐based obesity management, which is predominantly led by general practitioners (primary care) but may involve allied health (e.g., psychologists, dieticians, physiotherapists etc). Integration between community health services (such as allied health and primary care) and tertiary services is key in supporting patients in this transition period.^11^, ^13^ Australia's publicly funded health system, the Medicare Benefits Scheme (MBS) model, subsidizes five allied health visits to facilitate affordable outpatient multidisciplinary care for eligible patients.^14^ These services are available with a referral from a GP and may be used to access one allied health professional for five visits, or up to five different allied health professionals for one visit. There are ongoing questions surrounding the adequacy of this system to optimize outcomes in this population, due to accessibility and cost beyond the subsidized visits.^8^ The delivery of community‐based obesity management is known to be limited by the obesogenic environment, weight stigma, inadequate equipment/time, and lack of training around severe obesity in primary care.^15^ Exploration of patient perspectives on their own care journey can provide insights into the effectiveness of current models of care and highlight where improvements can be made to appropriately address their needs. Clinicians from tertiary WMPs can offer adjunctive insights into factors contributing to, or hindering, patient ‘success’ following discharge.

This study aimed to explore the needs of patients to facilitate their transition from tertiary WMPs to community‐based care and the provisions needed in community‐based care for those on waiting lists. Qualitative methods facilitated focus on clinician and patient experiences with community services, and potential needs for additional support.

## 2 METHODS

A qualitative study was undertaken to explore the experiences of patients and clinicians in a publicly funded hospital‐based tertiary WMP in Sydney, Australia.^16^ Semi‐structured interviews and focus groups were undertaken for data collection, conducted either face‐to‐face or via telephone, between December 2021 and November 2022. The consolidated criteria for reporting qualitative research (COREQ) was used as a framework for reporting (Appendix A).^17^ Ethics approval was granted by South Western Sydney Local Health District Human Research Ethics Committee approval number HREC 2019/STE00893.

### 2.1 Recruitment

Adult patients (aged >18 years) from the tertiary WMP with a BMI ≥40 kg/m^2^, and clinicians from the program including endocrinologists, nurses, psychologists, physiotherapists, and dieticians were purposively sampled. Patients recruited via the clinic staff were invited via an email, which explained the aims of the research and introduced the research team, to take part in a one‐on‐one interview. Clinicians were contacted by KAM (female, academic researcher) or JT (female, MD student) and invited to participate in a focus group or one‐on‐one interview. Informed consent was obtained either written or verbally. JT and KAM are both trained qualitative researchers (KAM PhD) and had no existing relationship with the patients though had a prior professional relationship with two of the clinicians.

### 2.2 Data collection

All one‐on‐one interviews were conducted via phone, and a focus group with clinicians was held face‐‐face at the clinic, with only participants and the interviewers present. The interviews and focus groups were performed by JT and KAM and were guided by either a patient or clinician interview schedule (Appendices B and C). Both focused on what supports were needed for successful community‐based care, especially during transition from the program, as well as experiences in the WMP versus community‐based care.

Individual interviews lasted ∼30 min and the focus group lasted ∼60 min, with field notes taken. No repeat interviews were held. A digital voice recorder captured the interview/focus group data before being transcribed using a secure service, NVivo.^18^ De‐identified transcripts were stored securely and were only accessible to JT and KAM. They were not returned to participants for review as we aimed for transcripts precisely reflecting what was said during data collection.^19^ The data collection ended after theoretical saturation was subjectively reached, as determined by the absence of new themes arising during data collection/coding.^20^

### 2.3 Data analysis and management

Thematic analysis approach was selected as it facilitates the interpretation through the identification of patterns in the data to develop themes and address research questions.^21^ Transcripts were uploaded to the Quirkos program where data was analyzed according to the principles of thematic analysis.^22^, ^23^ Two data coders (JT and KAM) were involved in data analysis, both independently coding the data before discussing and reflecting upon emerging themes. The data was revisited multiple times by both coders before the final coding report was generated using Quirkos.^23^

## 3 RESULTS

Fifteen patients were referred to the study, with 11 agreeing to participate. Two could not be reached, and two declined. Most participants in this study were female. Seven clinicians, primarily female allied health providers, were also contacted and consented to participate. The specific demographics of both groups can be seen in Tables 1 and 2.

**TABLE 1:** Demographic characteristics of patients.

Clinic patient characteristics	*n* = 11
Mean age (Std Dev)	54 (9.88)
Female	7 (63.64%)
Mean months in clinic (Std Dev)	24.88 (12.45)
No longer a patient of the clinic	1 (9.10%)
Has had bariatric surgery	3 (27.27%)
Currently part of a social support group	1 (9.10%)

**TABLE 2:** Demographic characteristics of clinicians.

Clinician^a^ characteristics	*n* = 7
Female	5 (71.43%)
Mean months in role (Std Dev)	49.29 (52.53)
Provider categories	
Allied health	4 (57.14%)
Endocrinologist	2 (28.57%)
Endocrinology trainee	1 (14.29%)

^a^ The term ‘clinician’ is used to refer to all healthcare providers within this study, including allied health providers, as they all provide clinical support to patients within this setting.

**FIGURE 1:**
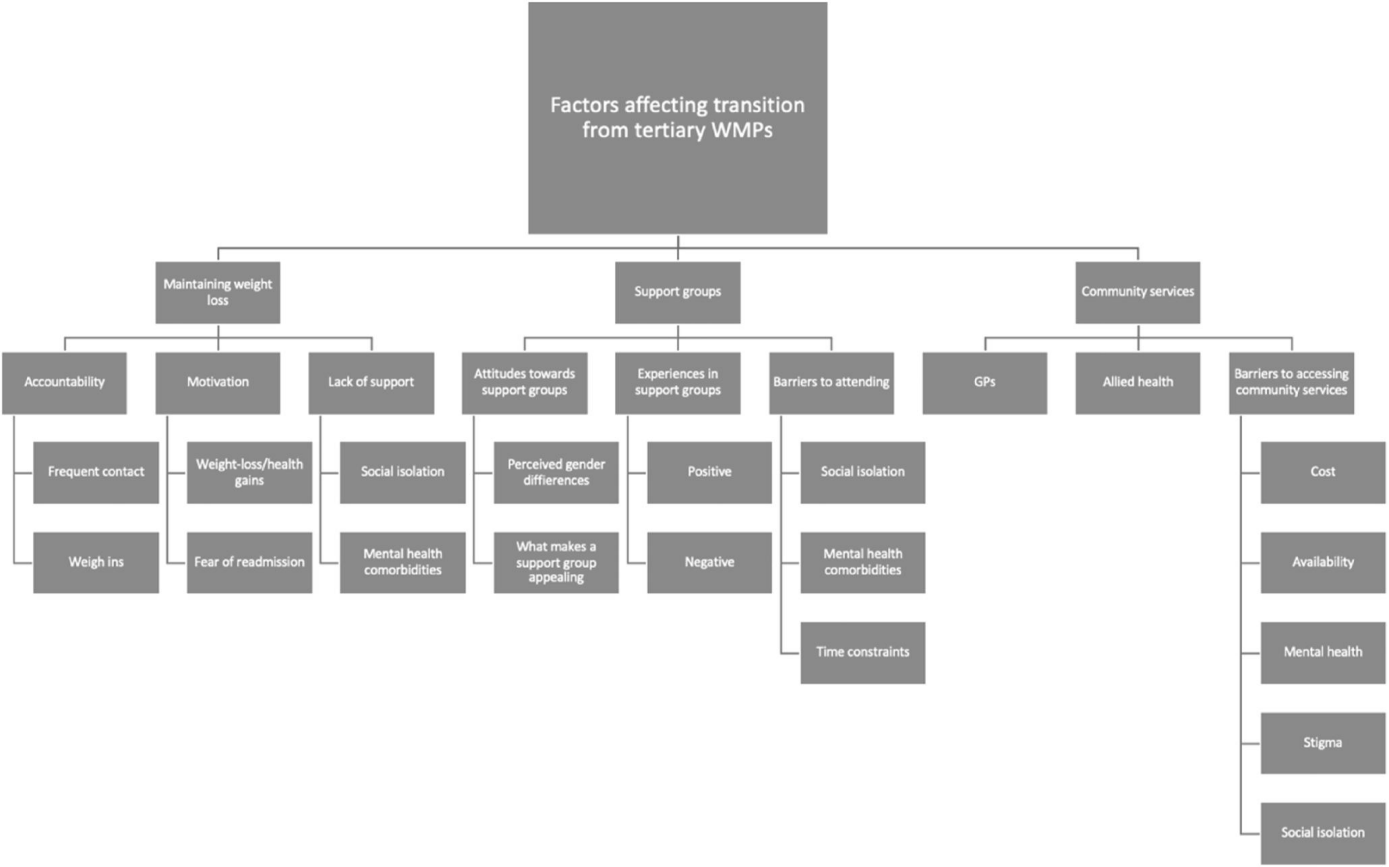
Coding tree used to define themes.

Data were classified into four major themes, each containing multiple subthemes^1^: The importance of accountability and motivation to maintain weight‐loss/health gains^2^; Limitations within community‐based obesity management for those with severe obesity^3^; Perspectives on structured community programs for patients transitioning into/out of tertiary WMPs^4^; Impact of mental health, stigma, and social isolation on engagement with community‐based services (Figure 1). Excerpts from interviews/focus group can be found in Tables 3, 4, 5.

**TABLE 3:** Importance of accountability and motivation to maintain weight‐loss/health gains.

Emerging theme	Excerpt no.	Excerpt
Importance of accountability for ongoing weight‐loss	1.1	“The one thing that we know that really makes a difference with people and weight‐loss is the regularity and frequency of contact.” (Clinician 6)
	1.2	“That's one thing that was helping me keep the weight off, knowing that I will be weighed next time, because the scale doesn't lie, and you go there and you feel that people are looking at what you are doing.” (Patient 11)
Difficulties with maintaining motivation	1.3	“When it comes to weight‐loss there's really not much to it. They say look at the nutrition, go on a shake or something. Do a bit of exercise and there you go, that's what you got to do. But to actually go and do that you need some sort of motivation.” (Patient 4)
	1.4	“You know, when you're sitting there and you basically feel like you're just eating fricking rabbit food and not losing any weight, you get really disheartened. It's like, why the hell am I doing this.” (Patient 6)
Isolation and lack of ongoing support	1.5	“You could put on a kilo or something and think, I don't feel good about myself, who am I going to talk to? And if you're finished with the clinic then what are you supposed to do?” (Patient 7)
	1.6	“I think people that have tried to lose weight for many, many years, know how hard it really is. They will lose some weight and then they put it straight back on because they don't have that support.” (Patient 8)

**TABLE 4:** Limitations within community‐based obesity management for those with severe obesity.

Emerging theme	Excerpt no.	Excerpt
Clinician perspectives on treating patients with obesity in the community	2.1	“At the moment the problem is, even if you have a good GP who knows what to do in terms of referring, that's not enough. If you've got somebody who has a BMI 50 kg/m^2^ and diabetes and you know they have to go to metabolic clinic, you do a referral and then they wait 2 years, and the GP isn't really getting much support from specialists either.” (Clinician 1)
	2.2	“Well from a physio perspective, I know a lot of my friends and other colleagues in the community are reluctant to treat obese patients given their comorbidities. They're a bit worried that if they push them too hard, for example, that something might happen. So, I can see that side of things. I've got the luxury of having a hospital, if something goes wrong you hit the MET call button and people come to the rescue. The other thing is equipment ‐ we're lucky the weight limits are so much higher here, but if you were to go to a standard gym their safe working limit would be like 150 kilos max.” (Clinician 2)
Patient attitudes toward general practitioners	2.3	“The GP doesn't do those kinds of appointments. The GP has thousands of sick people to look after, the clinic is just mainly to look after people like us.” (Patient 11)
	2.4	“I guess none of my GPs have ever had a weight problem and, some are quite understanding, but others don't work quite so well. My actual long‐term GP has moved to Queensland, so yeah, I'm really feeling in limbo right now because I haven't really connected with anyone else.” (Patient 2)
Missing services	2.5	“You probably need a couple of GPs or physicians or whatever that will coordinate the care for people with a bit more needs but not enough to get into this clinic. For here, you need to have a BMI 40 kg/m^2^ plus comorbidity; so, if you've got a BMI 40 kg/m^2^, you've got class 4 obesity, and you still can't get into this clinic. Plus, we are now at 450 people, so you are waiting at least 2 years, probably 3 years after covid, to be seen even if you qualify. So, you need a lot of care leading up to coming here.” (Clinician 1)
	2.6	“I think if people's local area health services had, you know, somewhere where you can go in and weigh‐in, reach out to some sort of support and services. If they can't do it at the clinic, then they could do it at your local community center.” (Patient 6)

**TABLE 5:** Perspectives on structured community programs for patients transitioning into/out of tertiary WMPs.

Emerging theme	Excerpt no.	Excerpt
Experiences with support groups	3.1	“In the past I'd go to weight Watchers and there was just that camaraderie, I guess. All of us being in various stages of transition through weight‐loss.” (Patient 3)
	3.2	“I ended up in hospital because the girls put me on a diet, but these girls were all wearing skin tight lurex tops and, you know, lycra pants. They had never been overweight in their life. And they put me onto this stuff, basically eating something like two boiled eggs a day. So, I ended up in hospital after passing out from not eating enough food for my weight. So, I just stopped going to all that sort of stuff.” (Patient 3)
Patient attitudes toward support groups	3.3	“I think that sort of thing helps you to keep on track. People who have gone through the same thing, or are going through the same thing, to be able to get together and go, ‘well this worked for me’ or ‘that didn't work for me’, and ‘how are you going?’. That just boosts you to keep going, that motivates you.” (Patient 6)
	3.4	“I had to wait, you know, a significant amount of time before I could get to the clinic. So, in that time I could have been being supported and I could have actually started the journey before, you know? And that type of thing. So yeah, I definitely think that (a support group) would have helped.” (Patient 8)
Perspectives on coordinating and structuring support groups	3.5	“I think if you had groups that had a multidisciplinary kind of supervision around them then that would increase their access to, you know, psychology, dietician, diabetes educator.” (Clinician 5)
	3.6	“I think so long as the support group is still run by or affiliated with health people, I think that would be good. I Think if you've got someone who is overseeing these meetings and they can have a guest speaker coming in and, you know, talk about certain issues or whatever, or a motivational speaker or something like that… I think it needs to be medically run if that makes sense.” (Patient 6)

### 3.1 The importance of accountability and motivation to maintain weight‐loss/health gains

Maintaining weight‐loss after leaving the WMP was a major concern, with patients and clinicians highlighting several factors that could contribute to success. Frequent contact with healthcare providers was highlighted by clinicians as an important determinant of sustained weight‐loss following discharge (excerpt 1.1). Furthermore, weigh‐ins and support/encouragement from WMP staff were reported by patients to contribute to increased accountability for maintaining weight‐loss (excerpt 1.2).

Maintaining motivation was a major challenge for most patients. Several patients expressed that fluctuating motivation could undermine their persistence with weight‐loss after leaving the clinic, even if part of group sessions (excerpt 1.3). Other participants expressed concerns regarding the longevity of their motivation to lose weight and explained how this related to their perceived success in weight‐loss so far (excerpts 1.4).

Patients were concerned that lack of support and social isolation would hinder sustainable weight‐loss outside the clinic. They highlighted the difficulty of trying to lose weight as a person with obesity and explained the relationship between success in this area and their need for support beyond the clinic (excerpts 1.5 and 1.6). Fear of relapse was mentioned by a few participants concerned about lack of community support and the likelihood that this would lead to weight re‐gain (Table 3).

### 3.2 Limitations within community‐based obesity management for those with severe obesity

Clinicians and patients highlighted many limitations within the management of patients with severe obesity in the community. Commentary on this topic was further separated into subthemes: clinician perspectives on community care, attitudes toward general practitioners (primary care), and ‘missing’ services.

Participating clinicians highlighted multiple issues in the community management of severe obesity. Physical accessibility to services—including provision of suitable bariatric equipment, for example, scales, and lack of support if something ‘goes wrong’—were highlighted as significant limitations (excerpt 2.1). According to one clinician, managing patients on clinic waitlists can also pose significant challenges to primary care providers who are often inadequately supported by specialists (excerpt 2.2). Further, one clinician stated that insufficient primary care education can hinder patient care and provider willingness to treat patients with severe obesity in the community (excerpt 2.1).

Many patients felt that their primary care provider has insufficient time to provide appropriate weight‐management support (2.3). Perceived lack of empathy for patients with obesity was also seen as a major barrier to engaging with primary care (excerpt 2.4). Many patients reported barriers to accessing the same GP consistently, therefore disrupting the continuity of care and being able to receive adequate community‐based support (excerpt 2.3 and 2.4).

Clinicians within the study expressed a need for more services to support patients before they became eligible for referral to WMPs (excerpt 2.5) or while they were on the waitlist. They highlighted that, during this time, community care for patients with severe obesity is highly inadequate (excerpt 2.5). One patient suggested that community centers could be leveraged as a venue for certain outreach activities such as regular/frequent weigh‐ins, which they believed would assist them in feeling supported (excerpt 2.6) (Table 4).

### 3.3 Perspectives on structured community programs for patients transitioning into/out of tertiary WMPs

Participant perspectives were also elicited with regard to support groups potentially being used to supplement community‐based obesity management. Subthemes included experiences in support groups, patient attitudes toward support groups and coordination/structuring of support groups.

Two of the 11 patients interviewed within this study reported experiences in support groups. One patient acknowledged that being part of a weight‐loss group gave her a sense of “camaraderie” and a common goal (excerpt 3.1). However, negative experiences within a later group had caused hesitancy toward joining any future support groups (excerpt 3.2). The benefits of being around similar individuals were echoed by a male participant, who stated that demographic differences including age can limit the extent to which participants can engage and support each other.

Most patients in this study described a degree of social isolation and expressed a desire for greater social connection and peer support. Additionally, patients believed that social isolation hindered weight‐loss by undermining their ability to maintain accountability and motivation. Several patients expressed interest in connecting with other individuals undergoing the same weight‐loss journey, and stated that a support group may help them overcome weight‐loss plateaus (excerpt 3.3). One participant highlighted the need for the support group model to be implemented for patients on the waitlist for tertiary centers, to provide social and weight management support *before* they gained access to the clinic (excerpt 3.4).

Many participants indicated that having health‐worker oversight for support groups would be important in coordinating group support. One clinician highlighted how multidisciplinary attendance to support groups could increase accessibility to appropriate information for patients who have left tertiary programs (excerpt 3.5). Consistent with this, other clinicians expressed concern that, without supervision, support groups may hinder patient success through the provision of conflicting/inaccurate advice. Most patients demonstrated interest in having an educational element to the group, with allied health workers (particularly dieticians) seen as being able to provide oversight and guidance during meetings (excerpt 3.6) (Table 5).

### 3.4 Impact of mental health, stigma, and social isolation on engagement with community‐based services

Most participants described mental health and weight‐stigma as significant obstacles to engaging with community‐based obesity support. Several patients identified that mental health comorbidities were major factors in deterring their attendance to any potential support groups, despite having an interest in seeking support for ongoing weight‐management (excerpts 4.1 and 4.2).

Many participants believed that obesity was managed and treated differently from other chronic health conditions, with blame and judgment often placed on patients (excerpt 4.3). One patient recounted an experience of attempting to seek support from her GP, whose insensitivity led to the patient's future reluctance to engage with medical services. Clinicians in the study acknowledged that stigma is often attached to having an obesity diagnosis and highlighted the ways in which GPs can fail to address the complexities of obesity pathogenesis.

Additionally, two clinicians highlighted challenges in providing support to male patients. One clinician identified that, with male patients, it is often difficult to provide direct emotional support. They suggested that activity‐based groups could be more successful in engaging male patients who may be avoidant of group situations requiring emotional vulnerability (excerpt 4.5). Furthermore, the ‘Men's Shed’ concept was mentioned as an example of an accessible community‐based group that provides targeted support to men. One male participant reported that discussing common interests is often a conduit for building deeper social connections and establishing a sense of group support (excerpt 4.6) (Table 6).

**TABLE 6:** Impact of mental health, stigma, and social isolation on engagement with community‐based services.

Emerging theme	Excerpt no.	Excerpt
Mental health as a barrier to accessing support	4.1	“I think that the number one hurdle for the group is that people don't want to talk about themselves because they're depressed and probably ashamed.” (Patient 1)
	4.2	“I'm all for groups and them getting together to help each other with everything, but like I said, I have pretty bad social anxiety. So, to me accessing it and actually using it are two different things. (Patient 5)
Stigma in the community	4.3	“It should be normalized that this is like a health condition like, you know, like diabetes or anything else. I mean, it's often said that if you were diagnosed with cancer, you wouldn't not treat it” (Clinician 6)
	4.4	“I go out and I think people are staring at me, I know I can feel it. They judge you because you're overweight, but they don't think that you might be sick or have an illness. There is a hell of a lot of stigma around, you know, it's just everybody's nature I think these days. I mean you try to do your best, you struggle with it.” (Patient 3)
Gender stereotypes affect how men engage with support groups	4.5	“So especially for men, if you say to a male, ‘Oh, look we've got this program, we'd like you to join a support group’ they'll say, ‘Oh, that's a bit fluffy I don't wanna do that’. But if you put them into an exercise program it becomes a bit like the Men's Shed concept where they're actually doing something and in the process of doing it they make friends and then they support each other.” (Clinician 7)
	4.6	“Traditionally, most blokes don't like talking about themselves. They prefer to talk about other things, whether it be the family, the car, or their job. But in those conversations what ends up happening is, if they talk to somebody for long enough, they eventually do ask ‘Are you all right?’ or ‘Do you need help’ or anything. Then they're more likely to say ‘Yeah, something's wrong’, or ‘Yeah I need the help’. Trust is a big thing for blokes.” (Patient 5)

## 4 DISCUSSION

Our study identified multiple barriers to maintaining weight‐loss/health gains for patients leaving tertiary (WMPs) when returning to the community setting. Our study addressed the widespread lack of research into the specific community‐based needs of individuals with severe obesity, and highlighted factors contributing to inadequacies within the current community‐based care model. While WMPs have been demonstrated to help patients attain a mean weight loss of around 6% at 12 months, weight regain is a major problem in this population.^24^, ^25^, ^26^ Patients and clinicians in our study expressed concern for the lack of targeted support available once patients have left tertiary programs, identifying the loss of regular support from the clinic as a significant barrier to maintaining weight loss. Weight‐stigma, co‐morbid mental health conditions, and social isolation have been reported to hinder engagement with existing community‐based services and/or support groups. Importantly, our results identified that patients waitlisted for tertiary programs are also likely to require targeted support while awaiting admission.

Our results indicate that the maintenance of weight‐loss and health gains is an ongoing challenge, with the pervasive nature of the obesogenic environment continuing to exert influence on patients even after ‘completing’ treatment in tertiary WMPs.^12^ This is a prominent limitation of community‐level care, and patients in our study expressed serious concerns about the abrupt loss of contact with the clinic. Additionally, they anticipated that this would detrimentally impact motivation and accountability for maintaining weight‐loss. Shared care between WMPs and primary care would likely facilitate more ‘economical’ contact with specialist weight management services. However, the literature on the management of obesity in Australia has identified major challenges to providing accessible and well‐integrated obesity care.^27^ This research highlights how the scarcity of multidisciplinary specialist obesity services and increasing rates of severe obesity have led to reduced accessibility to such services, with high patient load placing pressure on tertiary programs to discharge patients.^27^ Data collected from clinicians in our study reinforced the importance of regular clinic appointments and improved weight‐management/health outcomes. These findings are consistent with empirical literature which suggests a long‐term behavioral change for effective obesity management requires ongoing attention.^12^

Patients in our study believe that regular weigh‐ins contribute to the sustainable maintenance of weight‐loss. Regular weigh‐ins are controversial in obesity management due to the potential negative psychological impacts associated with failure to lose weight.^24^ Recently, literature has emerged in support of a shift toward a more ‘weight‐inclusive’ approach to obesity management, arguing that reducing the emphasis on weight will help mitigate the stigma and improve wellbeing in this population.^28^ Research has also identified a high prevalence of eating disorders among people with obesity, with previous data from this WMP demonstrating significant psychological distress and eating disorder risk among patients.^24^ This further complicates the potential use of regular weigh‐ins as a tool for maintaining accountability and weight‐loss.^29^, ^30^ While our findings suggest that weigh‐ins are an important part of maintaining weight loss, further investigation is required to better understand the needs of this population and balance physical and psychological health outcomes.^24^

Challenges in community‐based obesity management appear difficult to overcome within the current system. Empirical literature has established the difficulty of managing severe obesity in the community, with research continuing to recommend management in tertiary programs.^31^ Considering limited access to these services, our study defined the need for improved community‐based support for patients both *before and after* admission to tertiary WMPs. Data collected from clinicians in this study reinforces the complexity of managing obesity in the community, and highlights how time restrictions with patients, as well as limitations in training for the management of clinically severe obesity, can prevent GPs from adequately addressing patient needs. Similarly, there was a perception among patients that GPs are too busy to address weight‐related concerns. This is concerning, given the limited capacity of specialist WMPs in Australia and beyond.^2^, ^10^ These findings are echoed in the broader literature, which notes that short primary care consultations and insufficient counseling skills are major barriers to effective community‐based obesity care.^32^

Existing research has identified the need for improved primary care resourcing and training, with several studies highlighting the lack of provider confidence around identifying and counseling patients in need of obesity management.^32^ GP led WMPs must therefore play a role in alleviating pressure off tertiary obesity services, with trials like ‘The Change Program’ demonstrating early success in improving GP confidence in managing obesity.^33^ More broadly, research has found there are significant deficits in the current model of obesity management in Australia, which requires comprehensive improvements at all levels of the healthcare system.^27^ These findings identify alterations to funding, partnership with people living with obesity and the elimination of weight‐stigma, as key recommendations to improve obesity management.^27^ Research in the UK and Western Australia has introduced the concept of obesity ‘champions, ’ a term referring to non‐health professional/community peer‐support leaders.^34^, ^35^ Studies have suggested that champion‐led interventions are received positively by patients, and that champions may play a role in mitigating stigma and providing patients with local resources and social support.^35^ The application of this model in regions with a high burden of obesity may prove highly beneficial for patients transitioning into and out of tertiary WMPs.

Our study found widespread obesity stigma was a major barrier to engagement with community‐based services, contributing to social isolation and lack of support. These findings align with the literature on patient experiences of obesity, which highlights weight‐stigma as a significant barrier to engagement with health services.^36^ Inherent within weight‐stigma is the assumption that patients are to blame for having obesity, and that the reversal of obesity is a matter of personal responsibility.^15^ Literature has found that experiences of stigma are more prevalent among those with class 3 obesity compared to those with lower BMIs.^37^, ^38^

The stigmatization of obesity has led to widespread failure to address it as a chronic disease, contributing to many health issues experienced among individuals with obesity, such as disordered eating and depression/anxiety, as well as strong reluctance to engage with community‐based services.^24^, ^36^ In efforts to mitigate the stigma in healthcare, a recent report released by an Australian consumer organization, the Weight Issues Network, has led to calls for improved understanding from providers.^39^ Consistent and ongoing training of community‐based care providers is also urgently needed to reverse and mitigate obesity stigma.^32^, ^36^

Interestingly, patients within our study felt that without regular contact with the WMP, they would experience greater social isolation. Social isolation has been defined in the recent literature as “a sense of not belonging to society” and is notably distinct from loneliness (the subjective feeling of having too small a social network).^40^ Patients within this study felt isolated from society due to their high weight and widespread stigma. Furthermore, interplay between mental health comorbidities and the prevalence of weight‐stigma likely contributes to social isolation.^40^, ^41^ However, there is a scarcity of literature exploring the main drivers of social isolation within this population.^40^, ^41^ While the literature suggests an association between obesity and social isolation/loneliness, questions remain surrounding the cause and effect of this relationship.^41^ Patients in our study felt they were understood and treated without judgment during their time at the WMP, contributing to a reluctance to seek support in new, potentially stigmatizing, community environments following discharge. It is highly likely that patients who have not yet accessed tertiary multidisciplinary WMPs are also experiencing isolation and are subsequently under engaged in services as they wait to access such clinics. Interventions to mitigate social isolation in this population are needed to maximize engagement in support services, as the avoidance of health services is a known consequence of social isolation.^42^

Patients and clinicians within our study perceived support groups to be a potential means to access social support and weight‐management education on leaving WMPs. Patients felt that groups could provide a safe platform to connect with individuals undergoing a similar weight‐loss trajectory, which they believed would assist them in maintaining motivation and accountability. Peer support groups may therefore play a key role in reducing social isolation and weight‐stigma if the drivers of social isolation can be adequately investigated and addressed. Having an allied health presence could reduce the cost of accessing these services individually for patients once discharged from the clinic, with research supporting the use of group programs to combat the increased demand for healthcare providers.^27^ However, alterations to the Australian MBS model would be needed as the current system does not support group health delivery.^27^ Factors influencing patient engagement with support groups will no doubt vary greatly from person to person, identifying a potential need for support groups to be tailored toward different patient characteristics. The high prevalence of mental health comorbidities and social isolation/withdrawal among this group suggests that psychological support may be necessary to increase engagement and maximize the benefits that any support group can provide.^24^, ^40^

As with all qualitative studies, this research has limitations and is intended to outline focuses for future research directions. This study was conducted at a single tertiary level obesity center, which may have different characteristics from other specialist WMPs across the state. We acknowledge that patients who have undergone bariatric surgery may have differing support needs to patients who have not; however, we did not specifically interrogate this need, and have identified this as an area for further research. Furthermore, there are likely differences across patients from culturally and linguistically diverse populations, which may not be captured in this study because of the small sample size. Strengths of this study include the purposeful sampling of a range of clinicians and patients, as well as the diverse nature of patients to which the WMP caters.^24^

## 5 CONCLUSION

This study aimed to explore the experiences of patients and their clinicians as patients transition from tertiary WMPs to community‐based care. Qualitative methods captured clinician and patient perceptions of existing community‐based services, and the urgent need for additional structured support. There is currently inadequate support for patients leaving tertiary programs, with interventions needed to facilitate the maintenance of weight‐loss/health gains. Patients require targeted and integrated support to minimize the impacts of mental health comorbidities and social isolation, and to maximize wellbeing and service engagement. Structured support groups should be considered for patients waiting to access tertiary WMPs and as a means of facilitating transition once discharged. Future research should aim to establish how to provide these programs while addressing issues of social isolation and integrating with current models of tertiary and primary health care.

## CONFLICT OF INTEREST STATEMENT

The authors declare no conflicts of interest.

## APPENDIX B PATIENT INTERVIEW SCHEDULE

Interviewer to introduce themselves then remind interviewee of confidentiality, option to withdraw from the interview at any point and to ask for permission to record the interview.

### Demographic questions;

Age:

Highest education level:

Gender:

Postcode:

Existing clinic patient?

If yes for how long?

If not, on a waiting list or attending another clinic?

‐ How can you best be supported by the clinic when it is time for you to transition out of the service?
‐ What supports do you think you would need from outside the clinic to help you make this transition? *Prompts family, mental health services, general practice, allied health, support groups*
‐ What support groups, if any, have you been part of prior to this interview?

### If have taken part in a group:

‐ What do you think has contributed to the success of your group?
‐ How do you think these groups can be supported?
‐ What guiding principles do you think should apply to these groups (and new groups in the future)
‐ What do you think prevents others form joining in with these groups?

### If have not been part of a group:

‐ What has prevented you from taking art in a social support group?
‐ What would make these group more attractive to join?
‐ How do you think these groups can be supported (*prompt e.g., by the clinic, by the community)?*
‐ How do you think social support groups, like those connected to the Nepean Obesity Clinic can be integrated with primary care (e.g., GPs/allied health outside of the clinic) and/or with the clinic to help people like yourself transition out of the clinic?
‐ How do you think this framework (for example, social support groups, community‐based care integrated with support from the clinic could work for individuals who may not be able to access the obesity clinic?

## APPENDIX C CLINICIAN INTERVIEW SCHEDULE

Interviewer to introduce themselves then remind focus groups of confidentiality, option to withdraw from the focus group at any point and to ask for permission to record the interview.

### Demographic questions;

Age:

Gender:

Current role:

Time in role:

In this focus group we would like to examine the experiences you have had when caring for patients who have attended this clinical service and have been transitioned out of the service. Could you each please think about a specific patient and their transition and in turn, describe this experience to me?

### Prompts

‐ What do you think may have helped or hindered your patients during this time?
‐ How do you think the clinic can best support patients when it is their time to transition out of the service?
‐ What supports do you think your patients would need from outside the clinic to help you make this transition? *Prompts family, mental health services, general practice, allied health, support groups*

### Support groups

‐ What support groups, if any, have you helped facilitate of prior to this interview?
‐ What do you think contributed to the success (or failure) of that/those group/s?
‐ How do you see these successes being replicated in community‐based support groups?
‐ How do you think community‐based groups can be supported?
‐ What guiding principles do you think should apply to these groups (and new groups in the future)
‐ What do you think prevents individuals in the community from joining in with these groups?
‐ What would make these groups more attractive to join?
‐ How do you think social support groups can link with General Practitioners or the Camden Metabolic Rehabilitation Service to assist in transitioning to community care? Prompt: for example, for patients who have bene patients and individuals or have barriers to attending the clinic
